# Drinking water contamination potential and associated factors among households with under-five children in rural areas of Dessie Zuria District, Northeast Ethiopia

**DOI:** 10.3389/fpubh.2023.1199314

**Published:** 2023-06-09

**Authors:** Gete Berihun, Masresha Abebe, Seada Hassen, Adinew Gizeyatu, Leykun Berhanu, Daniel Teshome, Zebader Walle, Belay Desye, Birhanu Sewunet, Awoke Keleb

**Affiliations:** ^1^Department of Environmental Health, College of Medicine and Health Sciences, Wollo University, Dessie, Ethiopia; ^2^Department of Pre-clerkship, College of Medicine and Health Sciences, Wollo University, Dessie, Ethiopia; ^3^Department of Public Health, College of Health Sciences, Debre Tabor University, Gondar, Ethiopia

**Keywords:** water, contamination, fecal coliform, under-five children, Dessie Zuria district, Ethiopia

## Abstract

**Objective:**

More than half of the 700 million people worldwide who lack access to a safe water supply live in sub-Saharan Africa, including Ethiopia. Globally, approximately 2 billion people use drinking water sources that are contaminated with fecal matter. However, little is known about the relationship between fecal coliforms and determinants in drinking water. Therefore, the objective of this study was to investigate the potential for contamination of drinking water and its associated factors in households with children under 5 years of age in Dessie Zuria district in northeastern Ethiopia.

**Methods:**

The water laboratory was conducted based on the American Public Health Association guidelines for water and wastewater assessment using a membrane filtration technique. A structured and pre-tested questionnaire was used to identify factors associated with the potential for contamination of drinking water in 412 selected households. A binary logistic regression analysis was performed to determine the factors associated with the presence or absence of fecal coliforms in drinking water, with a 95% confidence interval (CI) and a value of *p* ≤ 0.05. The overall goodness of the model was tested using the Hosmer-Lemeshow test, and the model was fit.

**Results:**

A total of 241 (58.5%) households relied on unimproved water supply sources. In addition, approximately two-thirds 272 (66.0%) of the household water samples were positive for fecal coliform bacteria. Water storage duration ≥3 days (AOR = 4.632; 95% CI: 1.529–14.034), dipping method of water withdrawal from a water storage tank (AOR = 4.377; 95% CI: 1.382–7.171), uncovered water storage tank at control (AOR = 5.700; 95% CI: 2.017–31.189), lack of home-based water treatment (AOR = 4.822; 95% CI: 1.730–13.442), and unsafe household liquid waste disposal methods (AOR = 3.066; 95% CI: 1.706–8.735) were factors significantly associated with the presence of fecal contamination in drinking water.

**Conclusion:**

Fecal contamination of water was high. The duration of water storage, the method of water withdrawal from the storage container, covering of the water storage container, the presence of home-based water treatment, and the method of liquid waste disposal were factors for fecal contamination in drinking water. Therefore, health professionals should continuously educate the public on proper water use and water quality assessment.

## Introduction

Water is the second most important resource for the existence of all living things, including humans (1). Access to safe water supply and sanitation is recognized as a basic human right. It also plays a critical role in achieving adequate nutrition, gender equality, education, and poverty eradication (2–6). Water safety depends on a variety of factors, from the quality of the source water to its storage and handling in the home (7). More than half of the 700 million people worldwide who lack access to a safe water supply live in sub-Saharan African countries, including Ethiopia (8). In developing countries, more than 80% of the burden of disease is associated with poor drinking water quality, largely due to contamination from unsanitary conditions (8, 9).

Fecal coliforms are a group of bacteria from the normal flora of human and animal feces that can contaminate soil and water. *E. coli* in water sources is responsible for disease outbreaks (10). According to a World Health Organization report (WHO), an estimated 1.8 billion people rely on water contaminated with *Escherichia coli* (*E. coli*) (8). In rural areas of most developing countries, bacterial contamination of drinking water is a major cause of waterborne disease. Bacterial infections caused by contaminated drinking water remain a serious threat to public health, including fatal diarrhea. The problem is particularly severe in third World countries due to deteriorating environmental conditions caused by high levels of open defecation. Worldwide, 215 million people defecate in the open, which is a major source of diarrheal disease transmission in under five children, usually caused by *E. coli* (11).

According to WHO, unsafe water, poor sanitation, and inadequate hygiene cause 1.5 million preventable deaths each year, with children under five being the most affected populations. Eight million children die before they turn five each year, and diarrheal diseases cause 250 million missed school days (12). Diarrhea and waterborne diseases are the leading cause of mortality and morbidity in developing countries. Globally, 2 billion people use contaminated drinking water sources that are contaminated with feces (13, 14). More than 1.1 billion people in the world still live without access to safe drinking water sources, two-thirds of whom live in Africa, especially in southern and southern Africa. In addition, 2.4 billion people do not have access to even basic sanitation, resulting in 1.8 billion deaths each year from diarrheal diseases, mostly children under five.

In third world countries, 80% of all illnesses are due to poor quality drinking water, largely due to contamination from unsanitary conditions (8, 15). The major health problem in Ethiopia continues to be communicable diseases, which primarily affect the low socioeconomic status population (16). A lack of basic hygiene affects a child’s ability to survive, grow, and develop. Inadequate water, sanitation, and hygiene (WASH) is directly related to malnutrition and is associated with recurrent diarrheal diseases or intestinal worm infections (2). It still accounts for about 11% of global child mortality, although the number of deaths has decreased by one-third in the last decade, from 1.2 million in 2000 to 0.7 million in 2011 (13, 17).

The highest rates of this problem on the African continent are in Ethiopia, where 20% of the urban population and 80% of the rural population lack access to clean water (1). More than 60% of communicable diseases are primarily due to adverse environmental factors such as unsafe and inadequate water supply and poor hygiene and sanitation practices (1, 6, 17). Sources of drinking water contamination can range from source to fork (1, 6, 18). Despite various studies on water supply in Ethiopia, there is still a large gap in quantifying drinking water contamination and associated factors, especially in rural Ethiopia (12). It has been difficult to obtain published data on drinking water quality contamination potential and associated factors. Therefore, the objective of this study was to assess drinking water contamination potential and associated factors in households with children under 5 years of age in rural areas of Dessie Zuria district in northeastern Ethiopia.

## Materials and methods

### Study area

**Figure 1:**
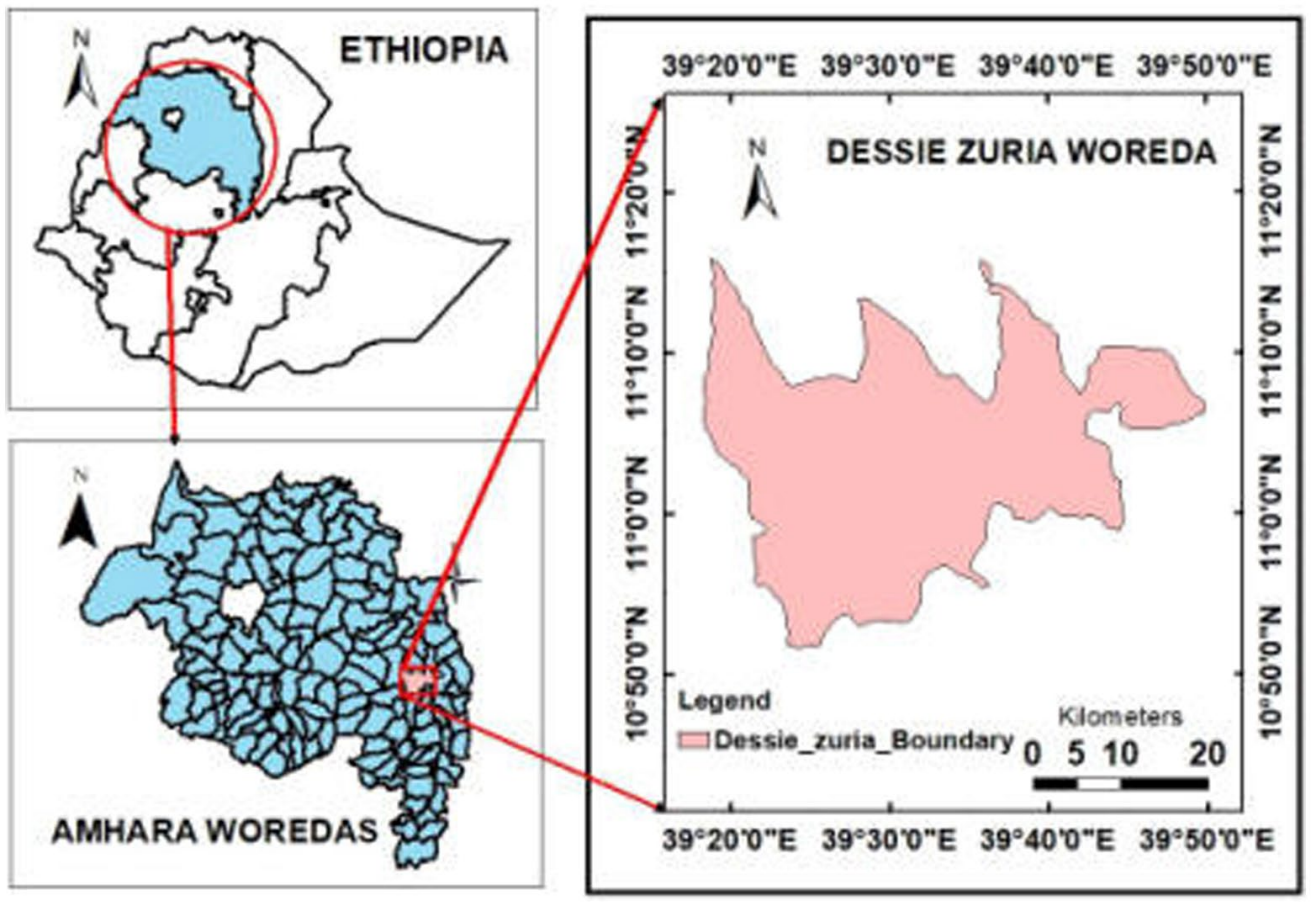
Map of Dessie Zuria district.

Dessie Zuria district is one of the 105 districts in the Amhara regional state of Ethiopia. It is located in the eastern parts of Ethiopian highlands in the southern Wollo zone. It borders Ilu to the south, Legambo to the west, Tenta to the northwest, Kutaber to the north, Tehuledere to the northeast, Kalu to the east, and Oromia zone to the southeast. It consists of 32 neighborhoods with a population density of 168.22, which is higher than the zone average of 147.58 persons per square kilometer (19). Based on the 2014 Ethiopian population projection report, the district has a total population of 176,309, of which 86,217 are males and 90,092 are females (20). A study conducted in Dessie Zuria district revealed that revealed that the prevalence of acute diarrhea among under-five children was 11% (95%CI: 7.8–14.3%) (21) (Figure 1).

### Study design and period

A community-based cross-sectional study was employed to assess the potential contamination of drinking water quality in households with children under 5 years of age. The study was conducted from January 1 to February 30, 2021.

### Populations of the study

#### Source population

The source population of the study was all households in the Dessie Zuria district with children under 5 years of age during the data collection.

#### Study population

The study population for this study was all selected neighborhoods in the Dessie Zuria district and all households in which children under-5 years of age living at the time of data collection.

### Eligibility criteria

#### Inclusion criteria

Households which are used for residential purpose is included in the study. Households that had been living in the study area for more than 6 months were included in the study. Furthermore, respondents who were 18 years and older at the time of data collection were included in the study.

#### Exclusion criteria

Respondents who were seriously ill or had other mental health problems that prevented us from obtaining reliable information were excluded from the study.

### Sample size determination

The sample size for the household survey was determined using the single population proportion formula (22) taking the assumptions that *p* (50%) of the household-level water samples are positive for fecal-contaminated drinking water, with a 95% confidence interval (CI) and a 5% margin of error.


N=Zα/22p(1−P)/D2

Hence, the sample size was 384 and considering a non-response rate of 10%, the final sample size of the study was 422. Before collecting the water samples, the survey data were collected from the selected households.

### Sampling procedure

Eight neighborhoods were selected from the total 32 neighborhoods by a simple random sampling technique using a lottery method. Then, the lists of households with children under 5 years of age from each selected neighborhood were obtained from the health extension workers of the selected neighborhoods. Then, the participant household from each neighborhoods was selected using systematic random sampling, using the *k*th value determined by dividing the study household by the total sample size. The number of participant from each neighborhood was determined by proportional allocation. *K* = the total number of households’/sample size 3,380/422 = 8.01 = 8.

### Data collection tools and techniques

The questionnaire was developed from the literature that had been published in an international reputable journal (23, 24) and modified according to the study setting. Water samples for fecal coliform determination were collected from household drinking water storage tanks. The water sample from each household was collected based on the American Public Health Association (APHA) guidelines for water and wastewater assessment (25). The sample was collected in a sterile 100-mL bottle container. To ensure the quality of the sample collected, the bottles containing the water samples were placed in a cooler with cold packs immediately after collection to maintain a temperature of 4°C and then transported to the laboratory for analysis within 24 h of sample collection (11, 25).

For the household survey, the data were collected using structured, pretested questionnaires. Initially, the questionnaire was prepared in English and translated into the local language (Amharic) and back-translated into English to assure its consistency. The data were collected through a face-to-face interview and observational. The questionnaire consists of the socio-demographic characteristics of the respondents, water handling practices, hygiene practices, sanitation, and waste management. A total of four Environmental Health professionals and two laboratory technicians were recruited to collect household data and water samples from January 1 to February 30, 2021. The supervision of the data collection process was done by two master’s holders of Environmental Health experts.

### Study variables

#### Dependent variable

The outcome variable of the study was the presence of fecal coliform contamination in a drinking water sample, with either (a yes/no) option.

#### Independent variables

The independent variables of the study were Sociodemographic characteristics (age, sex, marital status, family size, education level of the household head/spouse, occupational status of the household head/spouse, and monthly income); water handling practice; sanitation practices in the immediate area [safe disposal of human excreta (feces and urine) and disposal of household wastewater, proper segregation, collection, and disposal of solid waste, type of latrine, location of latrine, and ownership of latrine].

### Operational definitions

#### Fecal coliforms

Fecal coliforms are groups of thermos-tolerant, rod-shaped, non-spore-forming, Gram-negative, oxidase-negative, aerobic or facultative anaerobic bacteria capable of growing in the presence of bile salts or other surface-active substitutes with analogous growth-inhibitory activity and fermenting lactose with gas and acid (or aldehyde) production within 48 h at 44 ± 0.5°C (26).

#### Improved water source

Tap water in home, yard, or property, public tap or standpipe, tube well or borehole, protected dug well, protected springs, and rainwater collection (27).

#### Improved sanitation

Flush or drain to the sewer, septic tank or pit latrine, ventilated improved pit latrine (VIP), pit latrine with slab, and composting toilet (27).

#### Unimproved water source

Unprotected dug wells, unprotected springs, carts with small tanks or barrels, water supplied by tanker trucks, surface water (river, dam, lake, pond, stream, canal, and irrigation canal), and bottled water (27).

#### Unimproved sanitation

Flush or drain elsewhere (i.e., no piped sewer system, septic tank, or pit latrine), pit latrine without slab/open pit, bucket, hanging toilet or hanging latrine, communal facilities of any kind, and no facilities, bush or field (28).

#### Proper latrine use

Households with functioning latrines and at least no discernible feces on the premises, discernible fresh feces through the squat hole, and the footpath to the latrine were not covered with grasses (18).

#### Proper waste disposal

A method of disposal that included burning, burying in a pit, or keeping in a container and disposing of in a designated place (18).

#### Unsafe disposal of children’s feces

Unsafe disposal of children’s feces: disposal of children’s feces in open areas or not at all; feces is considered unsafe if left in the open, thrown in the trash, placed/washed/flushed down open drains, or buried (29).

#### Solid waste disposal

Disposing of waste by burning, burying it in a pit or storing it in a container, composting, and disposing of it in a designated location is considered “proper” disposal while disposing of waste in an open field is considered “improper” disposal (30).

#### Safe human excreta disposal

It is the practice of disposing excreta through better sanitation technologies such as pit latrine with slab, VIP latrine flush latrine and avoid of open defecation in the family members of the households (31).

#### Unsafe disposal of human excreta

It is the practice of disposing human excreta in unsanitary conditions like a pit latrine without slab, bucket, hanging toilet or hanging latrine, the absence of latrine facilities, or having the practice of excreting in bush or field (31).

### Data quality control

Data collectors and supervisors received 2 days training on the general aim of the study, the data collection process, and other relevant topics. The questionnaire was pretested on 5% of the sample size in Kalu district, who were excluded from the final study results. Necessary modifications were made based on the feedback from the pre-test before starting the final data collection. Water samples from each household were collected in sterilized glass bottles. All water samples were collected by trained laboratory technicians. Sample bottles were clearly labeled prior to sample collection. Samples were collected according to standardized procedures for collecting drinking water samples. All collected water samples were stored at 4°C prior to analysis. All water samples were analyzed in the laboratory within 4 h of collection. Prior to analysis, the required laboratory equipment and culture media were sterilized. All analytical procedures were carefully performed, and high-quality agar medium was used (17). Water samples were transported on ice in a cooler and analyzed within 2–4 h. In addition, the water samples were analyzed in triplicate according to the standard methods for the analysis of water and wastewater of the APHA guideline. The instruments were calibrated before use and throughout the process (25).

### Determination of fecal coliforms

A 100 mL volume of a water sample was drawn through a membrane filter (45-micron pore size) through the use of a vacuum pump. The filter was placed on a petri (culture) dish on a pad with lauryl sulfate broth media, which feeds coliform bacteria and inhibits the growth of other bacteria and incubated for 24 h at 44.50°C. This elevated temperature heat-shocks non-fecal bacteria and suppresses their growth. As the fecal coliform colonies grow, they produced an acid (by fermenting lactose) that reacts with the aniline dye in the agar, thus gave the colonies their blue color, and making them easier to count. After 22–26 h, the agar plates were removed from the 44.5°C incubator and counted the colonies that have any blue color. These were taken as positive for fecal coliform bacteria in the investigated water samples (25, 32).

### Data management and analysis

Epi Data version 3.1 and SPSS 25.0 were used for data entry and analysis, respectively. The results are presented in tables using frequencies and percentages. The outcome of the study was to measure the presence or absence of fecal coliforms in households stored for drinking water. A binary logistic regression analysis was performed to determine the factors associated with the potential for contamination of drinking water at the 95% confidence interval. Initially, a bivariable analysis was performed using a crude odd ratio (COR), and variables with a cutoff value of less than 0.25 were retained for multivariate analysis with a 95% confidence interval. In the multivariable analysis, the adjusted odds ratio (AOR) with the corresponding 95% confidence interval (CI) was then used to quantify the association between the dependent and independent variables with a 95% confidence interval. Therefore, variables with a value of *p* less than 0.05 at 95% CI were taken as factors significantly associated with the potential for contamination of drinking water by fecal coliforms. Analysis of fecal coliforms in drinking water was based on standard methods for water and wastewater testing adapted from APHA using the membrane filtration technique.

## Results

### Socio-demographic characteristics of the participants

In this study, 412 participants took part, with a response rate of 97.6%. The majority of participants were between 31 and 40 years old, Muslim, had primary education (1–8), were married, and were farmers with 178 (43.2%), 286 (69.4%), 123 (29.9%), 213 (51.7%), and 268 (65%), respectively. Nearly, half of the 186 (45.1%) households had an average family size of ≥5. Finally, half of the 213 participants (51.7%) had an average monthly household income of less than 1,000 Ethiopian Birr (Table 1).

**Table 1:** Socio-demographic characteristics of the respondents in Dessie Zuria district Northeastern Ethiopia from January to February 2021.

Variable	Category	Frequency (*n*)	Percentage (%)
Age (years)	18–30	154	37.4
	31–40	178	43.2
	41–50	80	19.4
Religion	Orthodox	111	26.9
	Muslim	286	69.4
	Protestant	15	3.6
Marital status	Married	213	51.7
	Widowed	114	27.7
	Divorced	85	20.6
Educational status	Cannot read and write	116	28.2
	Read and write	99	24.0
	Primary (1–8) grade	123	29.9
	Secondary (9–12) grade	50	12.1
	College and above	24	5.8
Occupation	Farmer	268	69.4
	Government employer	71	17.2
	Private business worker	43	10.4
	Housewife	30	7.3
Average family size	>5	186	45.1
	≤5	226	54.9
Ownership of the house	Own	286	69.4
	Rent	126	30.6
Average household monthly income	>3,000	95	23.1
	1,001–3,000	104	25.2
	0–1,000	213	51.7

### Water supply condition

The results of this study revealed that a quarter 103 (25.0%) of households used river water as their primary source of drinking water. Generally, unimproved water sources accounted for more than half of 241 (58.5%) of the households. More than half of 234 (56.8%) of the households’ primary sources of water supply water located at a distance of at least 1 km. The study also revealed that nearly two-thirds of 252 (61.2%) households placed their drinking water storage containers on the floor. Additionally, more than one-third [152 (36.9%)] of the households used the dipping method of water withdrawal from storage containers. Furthermore, one-third 162 (39.3%) of the households practiced home-based water treatment. Finally, this study also indicated that less than half of the 194 (47.1%) households had covered their household water storage containers during the inspection period (Table 2).

**Table 2:** Water supply conditions and handling practice in Dessie Zuria district Northeastern Ethiopia, from January to February 2021.

Variable	Category	Frequency (*n*)	Percentage (%)
Main sources of water supply	Tap water	49	11.9
	Protected spring	99	24.0
	Unprotected spring	96	23.3
	River	103	25.0
	Hand-dug wall	65	15.8
Types of water supply	Improved	171	41.5
	Unimproved	241	58.5
Alternate sources of water supply	Tap water	24	5.8
	Protected spring	78	18.9
	Unprotected spring	153	37.1
	River	116	28.2
	Hand-dug wall	41	10.0
Distance of water source from home	≤1 km	178	43.2
	>1 km	234	56.8
Round trip time to fetch water	>30 min	222	53.9
	≤ 30 min	190	46.1
Water consumption *per capita* per day	<20 liters	288	69.9
	≥ 20 liters	124	30.1
presence of water interruption	Yes	201	48.8
	No	211	51.2
Water storage duration	≥ 3 days	208	50.5
	< 3 days	204	49.5
Water collection container	Jerry cans	241	58.5
	Clay pots	109	26.5
	Buckets	62	15.0
Water storage container	Buckets	216	52.4
	Jerry cans	163	39.6
	Clay pots	33	8.0
Placement of water storage container	On the floor	252	61.2
	Elevated above the floor	160	37.8
Method of water withdrawal from the storage container	Pouring	260	63.1
	Dipping	152	36.9
Water container covered during the inspection	Yes	194	47.1
	No	218	52.9
Frequency of cleaning water storage container	Weekly	76	18.4
	Once per two weeks	189	45.9
	Always before fetching water	147	35.7
Presence of home-based water treatment	Yes	162	39.3
	No	250	60.7
Type of treatment	Boiling	35	21.6
	Filtration	85	52.5
	Chemical	30	18.5
	Solar disinfection	12	7.5

### Sanitation facilities and related condition of the households

Less than three-quarters 285 (69.2%) of the households had latrines during the survey time, of whom only 208 (73.0%) households used the latrine properly. Of the households which had latrines, half 143 (50.2%) of the households used pit latrines without slabs. Regarding the cleanliness of latrines, less than half of 129 (45.3%) were clean. This finding also indicated that less than half of 177 (43.0%) households had hand-washing facilities at home, and 127 (30.8%) households used only water for hand washing. Finally, this finding revealed that about two-thirds of 272 (66.0%) of the sampled drinking water was positive for fecal coliform (Table 3).

**Table 3:** Sanitation facilities conditions and related variables in Dessie Zuria district, northeastern Ethiopia, from January to February 2021.

Variables	Category	Frequency	Percentage
Availability of latrine	Yes	285	69.2
	No	127	30.8
If yes for the above question, what is the type of latrine?	VIP	36	12.6
	Pit latrine with a slab	106	37.2
	Pit latrine without a slab	143	50.2
Ownership of latrine	Private	215	75.4
	Shared	70	24.6
Latrine utilization	Yes	208	73.0
	No	77	27.0
Frequency of latrine cleaning in the past 2 weeks	Not cleaned	151	36.7
	At least once	134	53.3
Distance of latrine from home	<15 m	162	56.8
	≥15 m	123	43.2
The latrine pit hole had cover	Yes	85	29.8
	No	200	70.2
Cleanliness of latrine	Good	129	45.3
	Poor	156	54.6
Child feces disposal method	Safe	193	46.8
	Unsafe	213	53.2
Place of defecation in the absence of latrine	Open field	200	48.5
	Communal latrine	212	51.5
Solid waste disposal method	Safe	192	46.6
	Unsafe	200	53.4
Liquid waste disposal method	Safe	154	37.4
	Unsafe	258	62.6
Are their handwashing facilities in their home?	Yes	177	43.0
	No	235	57.0
Do you wash your hands after visiting toilets?	Yes	227	55.1
	No	185	44.9
What types of materials you use for handwashing?	Soap and water	193	46.8
	Ash and water	92	22.3
	Only water	127	30.8
Water samples positive for fecal coliform	Yes	272	66.0
	No	140	34.0

### Factors associated with fecal contamination of drinking water

In multivariable logistic regression analysis, duration of water storage in the house, method of water withdrawal from storage containers, covering of drinking water storage containers during the inspection, practice of household water treatment, and type of human excreta disposal were factors significantly associated with the potential for contamination drinking water at (95% CI). Drinking water stored for more than 3 days was 4,632 times more likely to be positive for fecal coliform than the corresponding groups. Households with the practice of dipping method of water withdrawal from storage containers were 4.377 times more likely to have fecal coliform bacteria than the corresponding groups. Furthermore, households which did not cover the water storage containers during the inspection time were 5.700 times more likely to have fecal coliform bacteria than the corresponding groups. Additionally, households which did not practice home-based water treatment were 4,822 times more likely to test positive for fecal coliform than the corresponding groups. Finally, households which had poor human excreta disposal practice were 3.066 times more likely to have positive for fecal coliform bacteria than the corresponding groups (Table 4).

**Table 4:** Factors associated with the contamination potential of drinking water and associated factors among households with under-five children in rural areas of Dessie Zuria, District, Northeast Ethiopia, from January to February 2021.

Variable		Positive for fecal coliform		COR (95% CI)	AOR (95% CI)	*p* value
		Yes	No			
Water storage duration in the past 2 weeks	≥ 3 days	172	36	4.969 (3.162–7.809)	4.632 (1.529–14.034)	**0.007** ^*^
	< 3 days	100	104	Ref	Ref	
Placement of drinking water storage container	On the floor	194	58	3.516 (2.295–5.388)	2.632 (0.974–7.101)	0.056
	Elevated above the floor	78	82	Ref	Ref	
Method of water withdrawal from the storage containers	Pouring	216	44	Ref	Ref	
	Dipping	55	96	8.416 (5.301–13.361)	4.377 (1.382–7.171)	**<0.001** ^*^
Drinking water container covered during inspection	Yes	82	112	Ref	Ref	
	No	190	28	9.628 (5.687–15.105)	5.700 (2.017–31.189)	**<0.001** ^*^
Frequency of cleaning water storage container	Weekly	49	27	2.108 (1.191–3.731)	2.781 (0.666–11.616)	0.161
	Once per two weeks	155	34	5.296 (3.235–8.670)	4.493 (1.492–13.530)	0.081
	Always before fetching water	68	79	Ref	Ref	
Practice of home-based water treatment	Yes	84	78	Ref	Ref	
	No	188	62	2.816 (1.848–4.290)	4.822 (1.730–13.442)	**0.003** ^*^
Latrine utilization	Yes	106	102	Ref	Ref	
	No	70	7	9.623 (4.225–21.917)	3.850 (0.429–8.942)	0.12
Frequency of latrine cleaning in the past 2 weeks	Not cleaned	114	37	3.578 (2.164–5.916)	3.593 (0.285–10.048)	0. 15
	At least once	62	72	Ref	Ref	
The latrine pit hole had cover	Yes	44	41	Ref	Ref	
	No	132	68	1.809 (1.079–3.031)	5.848 (0.737–19.681)	0. 401
Is their observed feces on the floor of the latrine?	Yes	88	41	1.659 (1.019–2.700)	1.119 (0.401–3.127)	0.830
	No	88	68	Ref	Ref	
Child feces disposal method	Safe	112	81	Ref	Ref	
	Unsafe	160	59	1.961 (1.297–2.965)	1.672 (0.634–4.407)	0.299
Liquid waste disposal method	Safe	69	85	Ref	Ref	
	Unsafe	203	55	4.547 (2.942–7.028)	3.066 (1.706–8.735)	**0.036**
Are their functional handwashing facilities in home?	Yes	96	81	Ref	Ref	
	No	59	176	2.517 (1.658–3.821)	0.475 (0.162–1.391)	0.174
What types of materials used for handwashing?	Soap and water	100	93	Ref	Ref	
	Ash and water	66	26	2.361 (1.383–4.029)	1.689 (0.437–6.535)	0.448
	Water only	106	21	4.694 (2.717–8.110)	1896 (0.612–5.878)	0.268

Ref, reference variable; The bold values indicate that these variables are factors significantly associated with the outcome variable of the study.

## Discussion

Access to safe water supply, adequate sanitation, and hygiene facilities are essential necessities of life. Yet, the problem is severe in rural parts of the developing countries, including Ethiopia (33). More than 60% of communicable diseases burden in Ethiopia are highly associated with poor environmental conditions. Hence, people continue to rely on unimproved water supply sources, which are highly susceptible to various types of contaminants (17). The use of microbial-contaminated water is thought to be the cause of between 10 and 20 million deaths annually and 250 million cases of sickness worldwide. This finding revealed that more than half (58.5%) of the households relied on unimproved sources of drinking water supply, which was higher than the findings in Ethiopia (43%) (34) and Kenya (17.2%) (35). Poor access to improved water sources leads to frequent disease outbreaks, which may account for up to 80% of health burdens mainly in developing countries (34, 36) which creates a significant financial and social burden such as school absenteeism and loss of productivity (34).

The finding of the current study showed that about two-thirds (66.0%) of the water samples taken at the household level indicated the contamination of drinking water were tested positive for fecal coliform, which was matched with the finding in Pakistan (37). On the other hand, the current finding was higher than the findings in Ethiopia (50.2%) (38), (39%) (34), (56.5%) (17), (33%) (39), (40%) (40), (37%) (41), Uganda (8.7%) (8), and Kenya (17.3%) (42). On the contrary, this finding was less than the findings in Ethiopia (72.6%) (17), (80%) (43), and (83.3%) (44). This high prevalence of fecal coliform bacteria in drinking water samples at the household level may not only originated from poor handling practice but also initially from the sources of water supply, mainly for households who relied on unimproved sources of water supply. Based on the WHO guidelines, drinking water must be free from fecal coliform bacteria to be fit for consumption (38). Therefore, the quality of water and its associated factors, its quality should be assessed not only from the household level but also at the sources for identifying key points of contamination and designing an appropriate intervention.

The use of improved source of water supply may not be guarantee for safe water quality at the consumption level. Therefore, good water handling practice should be done in addition to utilizing improved sources of water supply to overcome the burden of water related diseases, especially for children under the age of 5 (33). Less than half (47.1%) of the households covered the drinking water storage container during the survey, which was lower than the findings in Ethiopia (90.9%) (45), (92.5%) (18, 35), and, Sudan (91.7%) (46). The covering of water storage containers was one of the factors, which affect the potential for contamination of drinking water for fecal coliforms, which was consistent with the findings in Kenya (42).

Poor water handling practices are highly associated with post-contamination of drinking water (28, 47). The practice of dipping method of water withdrawal from storage containers may cause contamination of water despite using water sources from contamination at the sources of water supply (48). In addition, utensils used to withdraw water from storage containers and the hands of people handling the water are also common sources of water contamination (49). More than one-third (36.7%) of households had practiced the dipping method of withdrawing water from storage containers, which was lower than the results in Ethiopia (53.9%) (45) and Nigeria (58.8%) (50). In contrast, this result was higher than the results in Ethiopia (27%) (18). The method of water withdrawal from the storage container is one of the determining factors affecting fecal coliforms in water, which was confirmed by the study conducted in Kenya (42, 48). Contaminated drinking water at the point of collection may be attributed to various factors that may be at the source, transport, storage, or at the household handling practice (42). The possible rationale for this finding could be the fact that those who withdraw water from the storage containers by the dipping increase the risk of contamination. Hands of water handler may harbor various types of pathogenic microorganisms due to their poor hygienic practice.

Household water treatment plays an important role in improving drinking water quality. This type of treatment plays a particularly important role for households that depend on unimproved water supply sources, especially in developing countries. It has the potential to reduce the risk of diarrheal diseases by up to 61%. Boiling is the most common method of household water treatment in low-and middle-income countries but is not always practiced effectively (36). The results of the current study showed that only about one-third (39.3%) of the sampled households practiced household water treatment, which is consistent with studies from Ethiopia (44.1%) (45) and (34.3%) (51). On the other hand, the result of this study was lower than the studies conducted in Ethiopia 60% (30) but higher than the study conducted in Ethiopia (14%) (36), 2.8% (18, 52), 25.4% in Kenya (42), and Sudan (19.8%) (46). Based on the results of the current study, the presence of home-based water treatment is one of the factors affecting fecal coliform contamination in drinking water, which is consistent with the studies conducted in eastern Ethiopia (44) and Kenya (48).

Improper disposal of human excreta, such as construction of latrines near water sources and inadequate protection of water at the source, are considered major causes of fecal contamination. In most cases, households with exposed feces have high levels of microbial contamination. Human feces may contain a variety of pathogenic microorganisms, which cause diseases outbreaks such as typhoid fever, dysentery, cholera, and gastroenteritis (42). The results indicate that unsafe disposal of human excreta is another factor affecting fecal contamination of drinking water, which is consistent with studies conducted in Kenya (42) and Ethiopia (17).

## Limitations of the study

This study has certain limitations. Some of the data were collected through interviews and self-report, which means that some of the responses may have social desirability bias. The study used a cross-sectional design, which may make it difficult to establish a cause-and-effect relationship between the dependent and independent variables of the study. Due to the hotspot nature of this study, there may be seasonal variations in the bacteriological quality of the drinking water. Furthermore, since the water samples were taken only from household level, the status of fecal coliform bacterial contamination of drinking water at the sources were ignored which may be a confounding factor for the fecal coliform bacteria at the household level.

## Conclusion

The results indicate that household drinking water is significantly contaminated with fecal matter. Fecal coliform bacteria were detected in more than two-thirds of the water samples. The length of time water was stored in the household, the method of water withdrawal from storage tanks, the presence of domestic water treatment, and the method of disposal of human excreta were factors significantly related to the potential for contamination of drinking water. Therefore, the district health department should work to improve the quality of drinking water through hygiene education by promoting simple, acceptable, and cost-effective treatment methods and the use of narrow-mouth containers such as jerricans and bottles. In addition, the health and water supply authorities should regularly monitor the bacteriological quality of drinking water at the source, in distribution, and in households. Finally, future research should focus on the quality of drinking water from source to consumption, using other types of bacteria in addition to indicator bacteria.

## Data availability statement

The raw data supporting the conclusions of this article will be made available by the authors, without undue reservation.

## Ethics statement

Ethics approval was obtained from the institutional ethics committee of Wollo University, University of Medicine and Health Sciences, under ethical reference number CMHS/675/13. The relevant letters were also requested from the district administration. Before the start of data collection, participants were informed of the purpose of the study and confirmed the confidentiality of their information, which would be used exclusively for scientific research purposes. Participation in the study was completely voluntary, and the autonomy of the participants was respected. Participants were also informed that they had the unrestricted right to withdraw from the study at any time.

## Author contributions

GB, MA, SH, AG, and LB contributed to the conception and design of the study. GB, SH, DT, ZW, and BD conducted the investigation. GB, LB, MA, AK, BD, ZW, BS, and AG performed data management and analysis. GB, MA, DT, AG, LB, ZW, and BS wrote and edited the manuscript. All authors contributed to the article and approved the submitted version.

## Funding

This research project was sponsored by Wollo University with Grant No.: WU/6879 n-05/13.

## Conflict of interest

The authors declare that the research was conducted in the absence of any commercial or financial relationships that could be construed as a potential conflict of interest.

## Publisher’s note

All claims expressed in this article are solely those of the authors and do not necessarily represent those of their affiliated organizations, or those of the publisher, the editors and the reviewers. Any product that may be evaluated in this article, or claim that may be made by its manufacturer, is not guaranteed or endorsed by the publisher.

## Abbreviations

AOR: Adjusted odd ratio
APHA: American public health association
CI: Confidence interval
COR: Crude odd ratio
*E. coli*: *Escherichia coli*
SSA: Sub Saharan Africa
WHO: World Health Organization
