# Supplementary material for: Drinking water contamination potential and associated factors among households with under-five children in rural areas of Dessie Zuria District, Northeast Ethiopia
Source: Front Public Health. 2023 Jun 9;11:1199314. doi: 10.3389/fpubh.2023.1199314 (PMC10289289; doi:10.3389/fpubh.2023.1199314)
Supplement: Supplementary file 1 [file Data_Sheet_1.PDF]

Information sheet (English version)

Hello! My name is ____________________and I am from _____________________. I am a member of a data collector team on behalf the study conducted by who is a researcher in Wollo University. The aim of the study will be to assess the contamination potential of drinking water and associated factors in households in under-five children in Dessie Zuria district Northeast Ethiopia. It involves face to face interview, observation and water sample collection for quality assessment. The maximum time for the accomplishing of the data collection will be 15 minutes. All the information that you are going to provide me will remain confidential since your name and identification number will no write but only the code. For this reason, I kindly request you to give me your sincere and truthful answer. Your participation in this study is entirely based on your willingness and you have the right to refuse, withdraw from the study at any time. Participation or refusal to answer questions will have no effect on your life. If you have any uncertainty or question about the request or would like to know the results of this study you can contact the principal investigator, research supervisor.

Do you agree to participate in the study: 1. Yes 2. No (mark one of them for verbal consent) your signature_______________________ (if written consent)

CONSENT FORM:

I have read / understand the Participant Information Sheet. I have been given sufficient time to consider whether or not to participate in this study. I understand that taking part in this study is voluntary (my choice) and that I may withdraw from the study at any time without this explanation. I know who to contact if I have any questions about the study in general. I understand that my participation in this study is confidential. I have asked some questions and clarification has been given to me. I have given my informed consent freely to participate in the study, and I__________________ hereby to approve my agreement with my signature.

English version questionnaire

Part I:Socio-demographic characteristics of the respondent and the household		
**Code**	**Variables**	**Options**
101	Age of the Respondents	___________
102	Religion of the respondents	1. Orthodox<br>2. Muslim<br>3. Protestant<br>4. Others
103	Marital status of the respondents	1. Married<br>2. Widowed<br>3. Divorced
104	Educational status of the respondents	1. Cannot read and write<br>2. Read and wright<br>3. Primary (1-8) grade<br>4. Secondary (9-12) grade<br>5. College and above
105	Occupation of the respondents	1. Farmer<br>2. Government employer<br>3. Private business worker<br>4. Housewife
106	What is Family size of the household?	___________________
107	Who is the ownership of this house?	1. Own<br>2. Rent
108	What is the average monthly income of the household?	__________________
**Part II: Water supply conditions and handling practice**		
201	What is the main sources of water supply of the household?	1. Tap water<br>2. Protected spring

		3. Unprotected spring<br>4. River<br>5. Hand-dug well<br>6. Others
202	What is the alternatives sources of water supply due to the interruption sources of water sources?	1. Tap water<br>2. Protected spring<br>3. Unprotected spring<br>4. River<br>5. Hand-dug well<br>6. Others
203	What is the average distance of water supply from your home?	___________________________
204	What is the average round trip time to fetch water from the sources?	_____________________________
205	What is the average daily water consumption rate of the household (L/C/d?	__________________________
206	Is there any water interruption in your main sources of water supply?	1. Yes<br>2. No
207	For how long do you stored drinking water in water storage container in your house?	________________________
208	What types of utensils you utilized for collection of water from the sources?	1. Jerrycans<br>2. Clay pots<br>3. Buckets<br>4. Others
209	What types of utensils you used for storage of drinking water at your house?	1. Jerrycans<br>2. Clay pots<br>3. Buckets<br>4. Others specify
210	Where do you put the drinking water storage container in your household?	1. On the floor<br>2. Elevated above the floor
211	what is the method of water withdrawal from	1. Pouring

	the drinking water storage container in your household?	2. Dipping
212	Is the drinking water storage container is covered during inspection?	1. Yes<br>2. No
213	How often do you clean the drinking water storage container in your house?	______________________________
214	Is their home-based water treatment in your household?	1. Yes<br>2. No
215	If your response for question #214 is yes, what is the method of home-based water treatment you used in your house?	1. Boiling<br>2. Filtration<br>3. Chemical disinfection<br>4. Solar disinfection<br>5. Others specify
**Part III: Sanitation facilities conditions and related conditions**		
301	Is their latrine in your compound?	1. Yes<br>2. No
302	If your response for question 301 is yes, what is the types of latrine?	1. Ventilated Improved Pit latrine<br>2. pit latrine with a slab<br>3. Pit latrine without a slab<br>4. Others specify
303	Who is the ownership of the latrine?	1. Private<br>2. Shared
304	Do you utilize your latrines always?	1. Yes<br>2. No
305	How often do you clean the latrine in the past two weeks?	_________________________
306	What is the average distance of latrine from your home?	_______________________
307	Does the latrine pit hole had cover?	1. Yes

		2. No
308	What seems like the overall cleanliness of the latrine?	1. Good<br>2. Poor
309	What is the way of child face disposal method?	1. Safe<br>2. Unsafe
310	Where do you excrete your faces in the absence of latrine?	1. Open field<br>2. Communal latrine<br>3. Others specify
311	What is the overall disposal way of solid wastes in your house?	1. Safe<br>2. Unsafe
312	What is the overall disposal way liquid waste in your households?	1. Safe<br>2. Unsafe
313	Are their handwashing facilities in their home?	1. Yes<br>2. No
314	If the answer is yes for the above question, what types of materials do you use for handwashing?	1. Soap and water<br>2. Ash and water<br>3. Water only
315	Do you wash your hands after visiting toilets?	1. Yes<br>2. No
316	Does the water sample tested for Faecal coliform bacteria is positive?	1. Yes<br>2. No

Thank you very much for your participation
